# Digital Twins in Radiology

**DOI:** 10.3390/jcm11216553

**Published:** 2022-11-04

**Authors:** Filippo Pesapane, Anna Rotili, Silvia Penco, Luca Nicosia, Enrico Cassano

**Affiliations:** Breast Imaging Division, IEO European Institute of Oncology IRCCS, 20141 Milan, Italy

**Keywords:** digital twins, personalized medicine, digital devices, digital patients, artificial intelligence

## Abstract

A digital twin is a virtual model developed to accurately reflect a physical thing or a system. In radiology, a digital twin of a radiological device enables developers to test its characteristics, make alterations to the design or materials, and test the success or failure of the modifications in a virtual environment. Innovative technologies, such as AI and -omics sciences, may build virtual models for patients that are continuously adjustable based on live-tracked health/lifestyle parameters. Accordingly, healthcare could use digital twins to improve personalized medicine. Furthermore, the accumulation of digital twin models from real-world deployments will enable large cohorts of digital patients that may be used for virtual clinical trials and population studies. Through their further refinement, development, and application into clinical practice, digital twins could be crucial in the era of personalized medicine, revolutionizing how diseases are detected and managed. Although significant challenges remain in the development of digital twins, a structural modification to the current operating models is occurring, and radiologists can guide the introduction of such technology into healthcare.

## Highlights

1. Digital twins are virtual real-time models designed to accurately reflect systems or physical objects.
2. A digital twin may show an issue before it happens in its real counterpart.
3. Similar to the benefits of manufacturing, digital twins can revolutionize healthcare.
4. The digital twin of an organ allows clinicians to practice procedures in a simulated environment.
5. A digital patient could improve early diagnosis and support personalized treatment planning.

## 1. Introduction

Digital Twin is a set virtual information construct that construes a potential or actual physical manufactured product from the micro atomic level to the macro geometrical level. At its optimum, any information that can be obtained from inspecting a physically manufactured product can be dynamically obtained in real time from its virtual counterpart. Such virtual models are used to analyze performances, test improvements, simulate issues, and generate potential solutions, all with the goal of generating valuable observations that can then be applied back to the real counterpart.

## 2. What Is the Difference between a Conventional Simulation and a Digital Twin

Simulation is a process that uses digital models to replicate a system’s various processes. Although such technology has been around for some time, the digital twin exemplifies an important new take, as digital twins are richer for study than a conventional simulation.

Conventional simulations work in finite element analysis. In the real world, these tests are used, for example, to analyze the stress effects of external pressure on devices/products to enhance their design or features. Nowadays, to run an appropriate simulation, digitization is needed, and this involves the design of 2D or 3D models representing assets within a process or a product. The simulation is then run by introducing variables into the digital environment or interface. In its basic form, a digital twin is the digital representation of physical or non-physical processes, systems, or objects. The digital twin also integrates all data produced or associated with the process or system it mirrors, allowing the real-time transfer of data within its digital ecosystem, mirroring the data transfer that occurs in the real world. As a conventional simulation can only represent a digital replica of an object—for instance, a radiological device—in a specific moment, exactly as it is, when it comes to product development, developers are stripped from the possibility of testing new concepts due to the fact that the potential to recreate a specific outcome is not possible. On the contrary, a digital twin can extrapolate how an object, such as a radiological device will work in the future after all necessary updates (or structural changes) are implemented [1].

Definitely, the difference between the conventional simulation and the digital twin is broadly a matter of scale: while a simulation generally studies one distinct process, a digital twin can itself simulate any number of variables in order to study multiple processes [2]. Within this digital world, all types of simulation can be run hypothetically. By creating a digital twin, it is possible to have insights about how to improve operations and increase the efficiency of a specific object/device or even to discover an issue before it happens in its real-world counterpart.

## 3. Digital Twin in Radiology: A Digital Device

Although such technology is completely brand new compared to conventional systems that are capable of ordinary manufacturing simulations, digital twin technology has already proved its value. In manufacturing industries, including in radiological devices, nowadays, the link between the physical and virtual counterparts is essential, and much of the modern engineering design and development rely strongly on validated, high-fidelity computer models [3]. The introduction of digital twins could empower the development and improvement of objects and help industries make needed object refinements before starting production. Even after a new object has gone into production, digital twins can help mirror and monitor the entire manufacturing process [2]. In the US, the digital twin market was valued at USD 3.1 billion two years ago, and it is estimated to reach USD 48.2 billion by 2026 [4].

**Figure 1:**
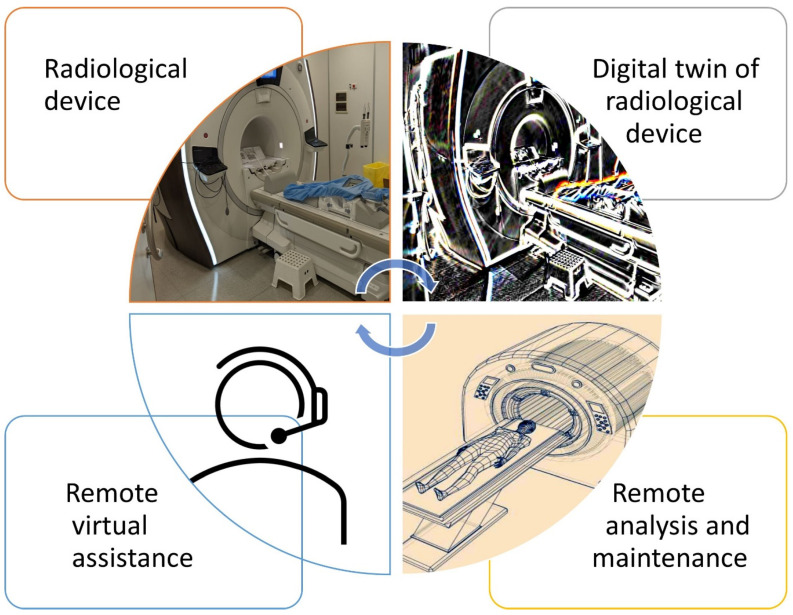
Key concepts and examples of radiological device digital twin life cycle.

Healthcare is one of the industries that could be deeply changed by the introduction of digital twins [5,6]. In radiology, digital twins of devices such as CT and MR machines (Figure 1) may allow the analysis of counterparts remotely and, in real-time, monitor their status, diagnosing issues, testing solutions, and even preventing problems before they occur thanks to artificial intelligence (AI) technology, which is crucial for both healthcare providers and patients to guarantee the continuity of care [7,8]. What the digital twin produces is a view of each device’s history and its potential future performance. This continuum of information can lead to early warnings, predictions, ideas for optimization, and, most importantly, a plan of action to keep devices in service for longer.

A digital twin of a radiological device is generated by sensors in a physical device. Data are relayed for remote analysis. To understand the data that a medical device transmits, intimate (human) knowledge of that device is required. Combining human knowledge and machine data (AI can also help detect patterns in data) means that a truly appropriate remote virtual assistance of the radiological device could be provided.

Digital twins of devices are not useful only for maintenance. They also facilitate the prototyping of innovative technology: for example, the agency NASA, or the international car racing Formula One, use digital twins in the design and development of vehicles, performing tests (including several iterations with physical prototypes) that otherwise would take years and building digital models that can be used to operate, simulate, and analyze an underlying system governed by physics [3,6,9].

The digital twin is a combination of data and intelligence that represent the structure, context, and behavior of a physical system of any type, offering an interface that allows one to understand past and present operations and make predictions about the future. Accordingly, such technology can help the organization of radiology departments, identifying ways to improve processes, upgrade patient experience, save operating costs, and enhance the value of care [1]. A digital twin may indeed be a four-dimensional model of a radiology department, whereby the cost and quality optimization parameters may be examined and ultimately selected based on the insights gained from simulations leveraging the digital twin [7].

## 4. Digital Twins in the Era of Personalized Medicine: From Digital Device to Digital Patient

The ultimate question is if digital twins offer so many advantages for maintaining and keeping medical/radiological devices appropriately working, is it possible to apply the same technology to humans? By integrating different measurements of a patient over time, is it possible to build a digital twin of a body part—such as an organ—and, finally, an integrated model of its anatomy and physiology?

Such digital organs may support radiology-guided therapy, where medical imaging is automatically integrated to guide interventional complex procedures. The education of young physicians and medical students would also benefit from this, allowing non-expert users to practice with digital patients [10].

Looking further ahead, if we integrated information on the enriched patient-specific models of the organ, we could predict diseases and better plan for the specific prevention (or treatment) of the single patient, reaching a truly personalized medicine [11]. If successful, it could enable the comprehensive digital twin tracking of an individual from birth to death. This could pave the way for a highly personalized diagnostics model capable of recommending lifestyle or dietary changes or even predicting upcoming diseases. This, in turn, could help find solutions to extend life through a healthier lifestyle or early intervention treatments [10].

Nowadays, with the availability of new technologies such as AI, we can already build personalized simulations for patients, continuously adjustable based on their tracked health/lifestyle parameters. Accordingly, there are already experimental digital twin models, which use real-time data to adjust treatments, monitor responses, and track lifestyle modifications [5,6,12,13,14]. The aggregation of digital twin models from real-world deployment will permit large cohorts of digital patients that may be analyzed for virtual clinical trials and population studies, providing more than individual patient predictions. The cumulative patient outcomes and the match/discordance between reality and predictions could produce inestimable evidence for research investment, channeling funds and energies into the therapies that show the most effectiveness [12]. Similarly, digital twins could help to structure existing radiology systems to better respond (in real-time) to patients’ needs and to unprecedented health situations and address health disparities as they occur [5].

**Figure 2:**
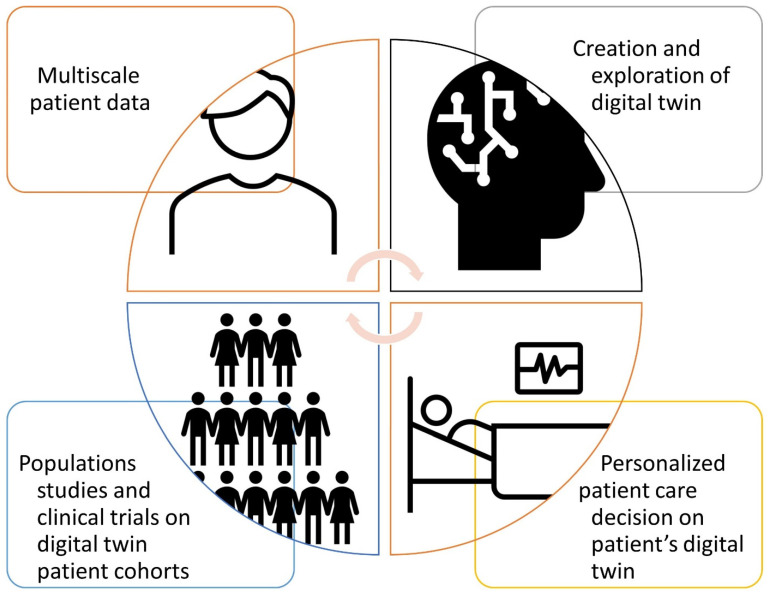
Key concepts and examples of patient digital twin life cycle.

Digital twins can create useful models based on the information obtained and recorded by wearable devices, creating a network linking patients, physicians, healthcare organizations, and drug and device industries [1,14]. With AI, innovative biotechnologies, and -omics measurements [8], in the near future, we may reach a lifelong, personalized model of a patient that is updated with any data and measurement of him/herself, including medical imaging, laboratory exams, and genetic data. This could be used to predict updated scenarios for various clinical changes, improve diagnosis, and support personalized treatment planning by targeted therapy delivery or providing lifestyle interventions tailored to the specific patient (Figure 2).

AI and -omics technologies explore models that create a digital twin of the patient. Medical decisions could be tested on the digital twin. The prediction of digital twins are based on virtual experiments, which could be integrated into the medical workflows for patient decision making and for constant learning-simulating clinical trials.

## 5. Current Limits and Challenges of Digital Twins Technology

Although the above-mentioned technologies are rapidly evolving, significant challenges remain in the development of digital twins, whose application in healthcare is still in a very early phase.

The first challenge concerns the generation and acquisition of high-volume, high-quality, validated, multiscale data that represent both healthy and diseased conditions. To create a digital twin, it is necessary to collect all kinds of data about the subject in large quantities and on a continuous basis. To ensure comprehensive data integration, data should be captured under FAIR (namely, findability, accessibility, interoperability, and reusability) principles and derived from diverse populations to ensure that every patient may equally benefit [5]. However, the variation in data that must be observed to create a model of an individual in a medical sense is extremely vast, and even the best current wearable devices are currently by far beneath notice.

In addition to the harmonization and aggregation of data, other challenges will include the development of multimodal data fusion methods that can combine knowledge to more accurately characterize disease information, the integration of data-driven and virtual modeling, and the abatement of model unpredictability via standardized training and validation [15].

Accordingly, as digital twins are affected by biases when learning from poor-quality data, careful controls and rigorous standards are required to ensure that they do not increase pre-existing biases and current inequalities in the healthcare system [5] and, at the same time, to regulate data governance and usage [12,16]. The applications of digital twins require gathering personal data by healthcare organizations and insurance companies, including the biological, genetic, physical, and lifestyle-related information of a person. Such individual data might be in use and benefit the company’s interests instead of the patients’ [5]. Digital twins’ technology has the potential to deliver significant societal benefits, but specific governance is demanded, including measures that ensure the transparency of data usage and derived benefits and data privacy [5,17]. Otherwise, instead of acting as a social equalizer by allowing for effective equalizing enhancement interventions, it could be a driver for inequality, given the fact that such technology could not be accessible to everyone and the fact that patterns identified across a population of digital twins could lead to segmentation and discrimination [5,16].

## 6. Conclusions

Although current data-driven methods are becoming more complex and intricate, digital twin innovation should adapt to the needs and workflows of physicians, helping them to better work with patients. Similarly to what happens with other innovations in modern medicine [8], the realization of digital twins in radiology and in healthcare, in general, can only succeed with interdisciplinary contributions of experts from various fields—from physicians to researchers—and different knowledge domains—from biology, medical science and physics to data science, image processing, computer science, and AI. Particularly, it would be arduous to develop a digital patient without the close involvement of radiologists who understand the way they look at images.

In the last few years, with the introduction of AI technology and the shifting of healthcare to a truly personalized medicine, a digital reinvention is already happening in radiology today, demanding true integration among the physical and digital views of assets, facilities, equipment, and processes. Digital twins will be a crucial part of that reformation, revolutionizing how diseases are detected, managed, and treated. The future of digital twins is full of potential, thanks to the increasing amounts of knowledge that are constantly being devoted to their use and their applications [4]. Radiologists, who always are at the forefront of the digital era in medicine [8], can guide the introduction of digital twin technology into healthcare.

## References

[1] Croatti A., Gabellini M., Montagna S., Ricci A. (2020). On the Integration of Agents and Digital Twins in Healthcare. J. Med. Syst..

[2] IBM.com (2021). What Is a Digital Twin?. https://www.ibm.com/topics/what-is-a-digital-twin#:~:text=A%20digital%20twin%20is%20a,reasoning%20to%20help%20decision%2Dmaking.

[3] Simonds S. Millions of Things Will Soon Have Digital Twins. https://www.economist.com/business/2017/07/13/millions-of-things-will-soon-have-digital-twins.

[4] Mehra A. Digital Twin Market Worth $48.2 Billion by 2026. https://www.marketsandmarkets.com/PressReleases/digital-twin.asp.

[5] Bruynseels K., Santoni de Sio F., van den Hoven J. (2018). Digital Twins in Health Care: Ethical Implications of an Emerging Engineering Paradigm. Front. Genet..

[6] Kamel Boulos M.N., Zhang P. (2021). Digital Twins: From Personalised Medicine to Precision Public Health. J. Pers. Med..

[7] Gossmann N. The Value of Digital Twin Technology. https://healthmanagement.org/c/hospital/whitepaper/the-value-of-digital-twin-technology.

[8] Pesapane F., Codari M., Sardanelli F. (2018). Artificial intelligence in medical imaging: Threat or opportunity? Radiologists again at the forefront of innovation in medicine. Eur. Radiol. Exp..

[9] Glaessgen E.H., Stargel D.S. The digital twin paradigm for future NASA and US Air Force vehicles. Proceedings of the 53rd AIAA/ASME/ASCE/AHS/ASC Structures, Structural Dynamics and Materials Conference.

[10] Martinez-Velazquez R.G.R., El Saddik A. Cardio Twin: A Digital Twin of the human heart running on the edge. Proceedings of the International Symposium on Medical Measurements and Applications (MeMeA).

[11] Hamlabadi K.G., Vahdati M., Saghiri A.M., Forestiero A. Digital Twins in cancer: State-of-the-art and open research. Proceedings of the Conference on Connected Health: Applications, Systems and Engineering Technologies (CHASE).

[12] Hernandez-Boussard T., Macklin P., Greenspan E.J., Gryshuk A.L., Stahlberg E., Syeda-Mahmood T., Shmulevich I. (2021). Digital twins for predictive oncology will be a paradigm shift for precision cancer care. Nat. Med..

[13] Rivera L.F., Jiménez M., Angara P., Villegas N.M., Tamura G., Müller H.A. Towards continuous monitoring in personalized healthcare through digital twins. Proceedings of the CASCON’19: Proceedings of the 29th Annual International Conference on Computer Science and Software Engineering.

[14] Hassani H., Huang X., MacFeely S. (2022). Impactful Digital Twin in the Healthcare Revolution. Big Data Cogn. Comput..

[15] Hussan J.R., Trew M.L., Hunter P.J. (2022). Simplifying the Process of Going From Cells to Tissues Using Statistical Mechanics. Front. Physiol..

[16] Pesapane F., Volonte C., Codari M., Sardanelli F. (2018). Artificial intelligence as a medical device in radiology: Ethical and regulatory issues in Europe and the United States. Insights Imaging.

[17] Zanca F., Brusasco C., Pesapane F., Kwade Z., Beckers R., Avanzo M. (2022). Regulatory Aspects of the Use of Artificial Intelligence Medical Software. Semin. Radiat. Oncol..

